# Muscle Extremely Low Frequency Magnetic Stimulation Eliminates the Effect of Fatigue on EEG-EMG Coherence during the Lateral Raise Task: A Pilot Quantitative Investigation

**DOI:** 10.1155/2018/7673068

**Published:** 2018-07-11

**Authors:** Qian Qiu, Liu Cao, Dongmei Hao, Lin Yang, Rajshree Hillstrom, Dingchang Zheng

**Affiliations:** ^1^College of Life Science and Bioengineering, Beijing University of Technology, Beijing 100024, China; ^2^Medical Engineering Research Group, Depart of Engineering and the Built Environment, Faculty of Science and Technology, Anglia Ruskin University, Chelmsford CM1 1SQ, UK; ^3^Department of Medical Science and Public Health, Faculty of Medical Science, Anglia Ruskin University, Chelmsford CM1 1SQ, UK

## Abstract

The aim of this study was to quantitatively investigate the effects of force load, muscle fatigue, and extremely low frequency (ELF) magnetic stimulation on electroencephalography- (EEG-) electromyography (EMG) coherence during right arm lateral raise task. Eighteen healthy male subjects were recruited. EEG and EMG signals were simultaneously recorded from each subject while three different loads (0, 1, and 3kg) were added on the forearm. ELF magnetic stimulation was applied to the subject's deltoid muscle between tasks during the resting period. Univariate ANOVA showed that all EEG-EMG coherence areas of C3, C4, CP5, and CP6 were not significantly affected by the force load (all p>0.05) and that muscle fatigue led to statistically significant reductions on the coherence area of gamma band in C3 (p=0.014) and CP5 (p=0.019). More interestingly, these statistically significant reductions disappeared with the application of muscle ELF magnetic stimulation, indicating its potential application to eliminate the effect of fatigue.

## 1. Introduction

Voluntary motor performance, as a result of the cortical command drive to muscle actions, is associated with the changes of characteristic oscillation and synchronization in the human sensorimotor cortex [1]. The electroencephalogram- (EEG-) electromyogram (EMG) coherence, representing the relationship between EEG (the recording of brain activity) and EMG (the recording of contracted muscle activity), is commonly used to examine a functional connection between human brain and muscles. It provides the mechanism information of the corticomuscular interconnection to better understand how a brain controls muscles [2, 3] and how different diseases, such as stroke [4, 5], tremor [6], and Parkinson's disease [7], lead to movement disorders [8].

It is well accepted that the physiological mechanism of corticomuscular coherence is not fully understood, but it is believed that the corticomuscular coherence between the brain and muscle activities is associated with the force loads and the modulation of fatigue [9–11]. EEG-EMG coherence has been used to quantify the functional corticomuscular coupling at different contraction levels during unilateral/bilateral motor tasks [12–14]. It has been reported that different force level influenced the electrical activities of related muscles and excitability of cortical areas [15, 16] and the EEG-EMG coherence [17]. However, the handgrip task performed in [17] is not easy to perform for stoke patients in real practice. It would be clinically useful to explore other alternatives. It has been known that, similar to the handgrip, the side arm lateral raise task would also lead to isometric contraction. However, the effect on EEG-EMG coherence with different force loads on the forearm during the arm lateral raise task has not been comprehensively quantified.

Muscle fatigue is described as a failure or a reduction in the capacity to maintain the expected force or power output after prolonged or repeated muscle contractions [18]. Fatigue-induced drop in motoneuron excitability with sustained muscle activity reduces the firing rate of active motor units and leads to significant weakening of corticomuscular coupling [8, 19, 20] or even neuromuscular diseases [5, 8, 9]. Although the influence of muscle fatigue on neuromuscular transmission or the functional coupling between brain and muscles has not been fully understood, it is hypothesized that the EEG-EMG coherence would change with muscle fatigue during the lateral raise task. Quantifying the difference in EEG-EMG coherence between fatigue and nonfatigue could provide scientific evidence to better understand the neural mechanism relating to muscle fatigue. This information could be used to develop treatment for different fatigue-related symptoms.

It is known that muscle magnetic stimulation can influence the activation of both cerebral cortex and muscle tissue. Peripheral magnetic stimulation (PMS) activates deep conductive structures and produces strong muscle contractions and massive proprioceptive afferents [21] and thus produces influences on the state of cortical excitability [22, 23]. Therefore, we hypothesize that this effect could be quantified by the EEG-EMG coherence. Although it is still controversial in the literature regarding the afferents recruited by PMS, when PMS is applied to the muscle indirectly, it is generally accepted that PMS activates mechanoreceptors via stimulation induced rhythmic contractions and relaxations and via muscle vibration. In addition, it also activates sensorimotor nerve fibers and could potentially modify the integrity of neuromuscular propagation [24]. Previous study has reported an enhancement in cortical excitatory of neurotransmission with pulsed and extremely low frequency (ELF) magnetic stimulation [25]. Although no specific parameters have been indicated to interfere with fatigue, previous studies have reported that ELF pulsed magnetic stimulation could induce neurofeedback [26] and facilitate reorganization of abnormal neural circuits and correct behavioral deficits [27]. However, the effectiveness of applying peripheral afferent magnetic stimulation has not been fully investigated on human subjects. Therefore, an investigation on the effect of the magnetic stimulation applied to deltoid muscle with EEG-EMG coherence could provide scientific evidence to support its potential clinical application.

The aims of this study were to quantitatively investigate the effects of force load and muscle fatigue on the EEG-EMG coherence in different frequency bands during side arm lateral raise tasks, as well as their different effects with the application of muscle ELF magnetic stimulation.

## 2. Materials and Methods

### 2.1. Subjects

This study recruited 18 healthy male subjects (right-handed, aged 25±3 years) who had no history of neurological or psychiatric disorders. The study was approved by the Local Ethics Committee of Beijing University of Technology and was conducted strictly according to the Declaration of Helsinki (1989) of the World Medical Association. The subjects were asked to sign a consent after being informed of the aims, potential benefits and risks of the study.

### 2.2. Experimental Procedure

**Figure 1:**
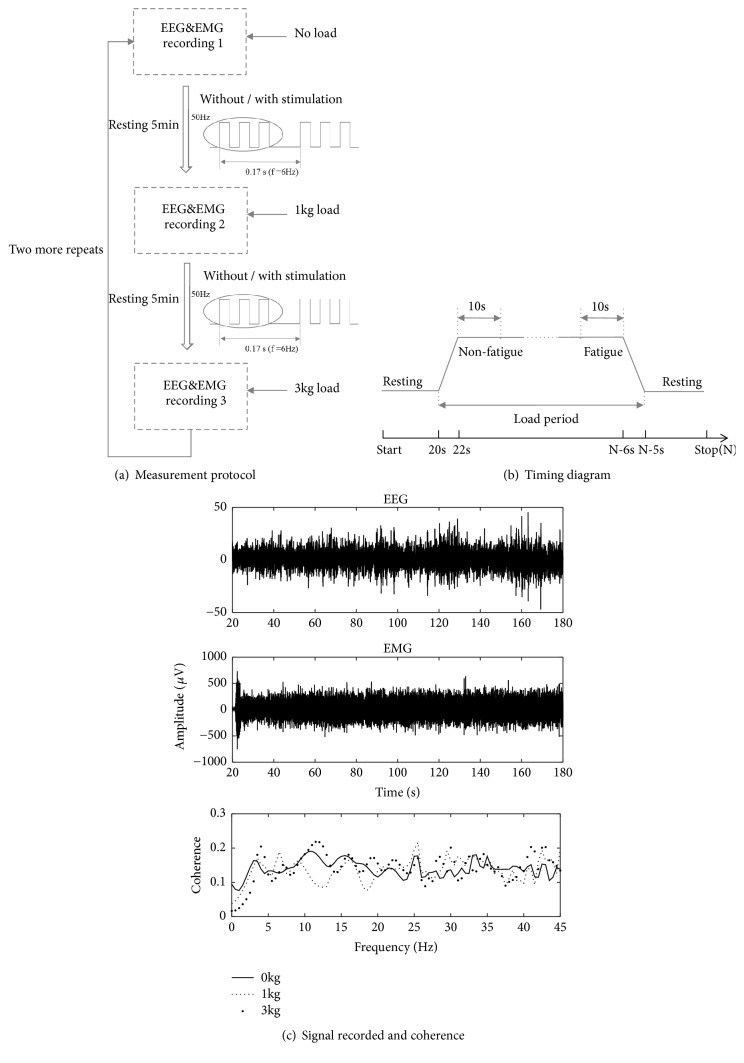
Measurement protocol (a), definition of fatigue and nonfatigue periods (b), and two examples of recorded EEG and EMG signals and their coherence (c).

To conduct the right arm lateral raise task, each subject was asked to sit comfortably with his right arm raised laterally (90 degrees away from the body) until he was exhausted. As illustrated in Figure 1(a), three different force loads (0 kg, 1 kg, and 3 kg) were added on the forearm in a randomized order between subjects to generate the isometric force at the upper limb muscle. The subjects had a five-minute rest between two consecutive tasks with different loads. Two days later, the same experiment was repeatedly performed on all the subjects, but this time the ELF magnetic stimulation was applied to the subject's deltoid muscle from a bespoke ELF magnetic stimulation device developed in our lab during the five-minute resting period. The intensity and frequency of the ELF stimulation were 30 mT and 6 Hz, respectively. EEG and EMG signals were collected from the same subjects and experimental sessions as the previous papers [15, 16].

### 2.3. EEG and EMG Recordings

32-channel EEGs and one-channel EMG were simultaneously recorded from each subject while the arm was laterally raised. The recordings continued until the subject was exhausted. The pin-type active-electrodes mounted in a headcap were applied on the head, and a BioSemi ActiveTwo (BioSemi, Netherlands) system was used for the EEG recording with a sampling frequency of 2048 Hz and 24-bit A/D resolution. During recording, common mode sense active electrode and driven right leg passive electrode were used as ground electrodes. The EMG signal was recorded from a pair of flat-tape active-electrodes placed on the anterior deltoid. There were a total of 18 EEG and 18 EMG recordings from each subject (9 from three force loads and three repeats without ELF magnetic stimulation and 9 from the repeated study with ELF magnetic stimulation).

### 2.4. EEG and EMG Signals Preprocessing

It has been generally accepted that the electrodes C3 and CP5 on the left hemisphere and C4 and CP6 on the right hemisphere over the brain have close relationship with the motor control, including the primary motor, sensorimotor, and parietal cortex [16]. Therefore, only the EEG signals from these four electrodes were further analyzed in this study [28, 29]. The first 10 s (from the start when the right arm was raised) of EEG and EMG recordings from each of the three force loads (0 kg, 1 kg and 3 kg) was regarded as nonfatigue status, and the last 10s recording before the subject was exhausted was regarded as fatigue status, as shown in Figure 1(b). The two segments of 10 s EEG and EMG signals were extracted, respectively, for each force load.  Figure 1(c) gives an example of the recorded EEG and EMG signals.

General noise was firstly removed from the EEG signals with a 0.5~45 Hz band-pass filter since EEG signal mainly includes alpha (7~13 Hz), beta (13~30 Hz) and gamma (30~45 Hz) bands. Next, the independent component analysis was applied to remove the noise caused by the blinks and eye movements, and the current source density transformation was applied to reduce the effect of volume conduction on EEG signals. For the EMG signals, the interference signals were removed with a 1~300 Hz band-pass filter and a 50 notch filter [30].

### 2.5. EEG-EMG Coherence

The coherence spectrum between the EEG and EMG signals provides a measure of their correlation in the frequency domain [31]. For each subject, the EEG-EMG coherence spectrum was calculated for each frequency bin of interest, as defined by the relation [17]

(1) $$\begin{array}{c} Coh_{xy}\left(\;f\;\right)=\frac{{\left|P_{xy}\left(f\right)\right|}^{2}}{P_{xx}\left(f\right)P_{yy}\left(f\right)} \end{array}$$

where *Coh*_*xy*_ is the coherence estimate of two signals *x* (EEG) and *y* (EMG) within different frequencies. The value of *Coh*_*xy*_ ranges from 0 (no coherence) to 1 (maximal coherence). *P*_*xx*_(*f*) and *P*_*yy*_(*f*) are the power spectral densities of signal *x* and *y*, respectively, and *P*_*xy*_(*f*) is the cross power spectral density of signal *x* (EEG) and *y* (EMG), as given by

(2) $$\begin{array}{c} P_{xy}\left(\;f\;\right)=\overset{\infty }{\underset{-\infty }{\sum }}R_{xy}\left(\;m\;\right)e^{-jfm} \end{array}$$

where *P*_*xy*_(*f*) is the Fourier coefficient of cross-correlation sequence *R*_*xy*_.

It has been reported that the coherence spectra at beta (13~30 Hz) and gamma (30~45 Hz) frequency bands were more prominent with voluntary contraction [20, 25]. Therefore, only the coherence areas at the two frequency bands were calculated using

(3) $$\begin{array}{c} S\left(\;f_{1},f_{2}\;\right)=\overset{f_{2}}{\underset{f_{1}}{\int }}Coh\left(\;f\;\right)df \end{array}$$

where *S*(*f*_1_, *f*_2_) is the coherence area within the frequency band [*f*_1_, *f*_2_] and *Coh*(*f*) is the coherence at *f*. Next, EEG-EMG coherence spectra of C3 and CP5 on the left hemisphere and C4 and CP6 on the right hemisphere were plotted individually for each subject to obtain their EEG-EMG coherence areas at both beta and gamma frequency bands.  Figure 1(c) gives one example.

### 2.6. Data and Statistical Analysis

The mean and standard deviation (SD) of EEG-EMG coherence area with different frequency bands were calculated separately for the three different force loads, for the fatigue/nonfatigue status, and with/without ELF stimulation. Univariate ANOVA analysis was then performed using software SPSS 23 (SPSS Inc.) to assess the repeatability between the three repeats within the same session and the effect of force load, muscle fatigue, and ELF magnetic stimulation on the averaging EEG-EMG coherence area of the three repetitions. A p-value below 0.05 was considered statistically significant.

## 3. Results

### 3.1. Effect of Force Load and Muscle Fatigue on EEG-EMG Coherence Area

Univariate ANOVA analyses showed that there were no significant differences between the three repeated measurements for all the coherence areas of C3, CP5, C4, and CP6 at different frequency bands (all p>0.05), demonstrating the reliability of this experiment. Therefore, their average values from the three repeated measurements for each force load was calculated as reference values for each subject, which were used for further statistical analysis.  Table 1 gives mean and standard deviation of EEG-EMG coherence area of different electrodes, separately for different force loads, for the fatigue/nonfatigue status, and with/without ELF stimulation. The data was presented as mean±SD.

Table 2 gives the statistical significant values from univariate ANOVA analysis, separately for the effects of force loads, for fatigue/nonfatigue status, and with/without ELF stimulation on the EEG-EMG coherence area. It indicates that all the EEG-EMG coherence areas from different EEG electrodes and frequency bands were not significantly affected by force load (all p>0.05). However, muscle fatigue had a statistically significant effect on the coherence area in C3 (p=0.006) and CP5 (p=0.046) within gamma band.

### 3.2. ELF Magnetic Stimulation Eliminated the Effect of Muscle Fatigue on Coherence Area

**Figure 2:**
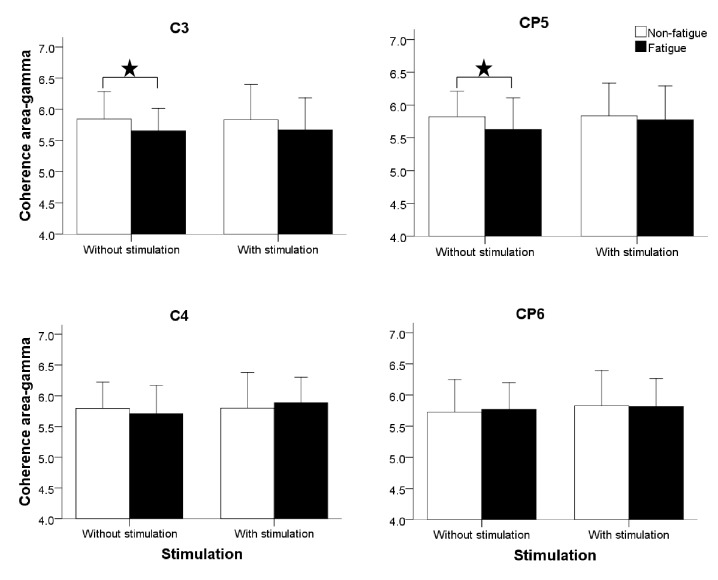
Comparison of the averaged EEG-EMG coherence area of force load 0, 1, and 3 Kg in gamma bands between fatigue and nonfatigue status. This was separately plotted for C3, CP5, C4, and CP6 electrodes and with and without ELF stimulation.

Figure 2 shows that, without ELF stimulation, the EEG-EMG coherence areas of gamma band from both C3 and CP5 showed statistically significant reduction in muscle fatigue status (p=0.014, p=0.019 were obtained from univariate ANOVA) in comparison with that from the nonfatigue status. With ELF stimulation, those statistically significant reductions with fatigue in gamma band from C3 and CP5 disappeared, and there were no statistically significant differences between nonfatigue and fatigue status with ELF stimulation (all p>0.05). For the EEG-EMG coherence areas of gamma band from both C4 and CP6, there were no statistically significant differences between fatigue and nonfatigue status, no matter whether ELF stimulation was applied between lateral raise tasks. There was no interaction between fatigue and ELF stimulation (all p>0.05).

## 4. Discussion

This study quantitatively investigated the effects of different force loads on the forearm and muscle fatigue status on the EEG-EMG coherence during the side arm lateral raise task and their effects with the application of muscle ELF stimulation between tasks. Without ELF stimulation, fatigue resulted in statistically significant reduction of the coherence area in gamma bands from C3 and CP5 electrodes. With the application of ELF stimulation, reductions of these coherence areas with fatigue were eliminated.

To investigate the effect of force load on EEG-EMG coherence, isometric force was generated with three different loads (0 kg, 1 kg, and 3 kg) on the forearm. Our results showed that there was no difference in EEG-EMG coherence area between different force loads whether fatigue/nonfatigue status and with/without ELF magnetic stimulation. One previous study investigated the shift of EEG-EMG coherence from beta band to gamma band with increased intensity of isometric voluntary contraction in tibialis anterior, but similar changes of EEG-EMG coherence with increasing contraction level have not been observed on soleus muscles [14]. Another study reported that corticomuscular coherence at 15~45 Hz increased significantly with the force level [32]. One possible explanation for the different results between our study and those of published studies lies in the different type motor tasks. In the above two studies, dorsiflexion/plantar flexion and finger movement were used to perform at different contraction levels. The other possible reason is associated with the applied force level. The increase of force from 0 kg to 3 kg in our study may not be able to recruit additional neurons and thus lead to statistically significant change of EEG-EMG coherence.

For the effect of muscle fatigue on EEG-EMG coherence, the results were affected by the application of ELF magnetic stimulation. Without ELF stimulation, the coherence area of gamma band in C3 and CP5 at fatigue showed statistically significant reduction with muscle fatigue. This agreed with published studies that found a decreasing tendency of EEG-EMG coherence with the development of the fatigue stage [8, 28, 33]. These statistically significant changes could be associated with the weakening of functional corticomuscular coupling, in which the inhibitory capacity to the descending motor pathway is strengthened or the neuromuscular junction transmission function decreased due to the muscle fatigue [8, 34, 35]. Additionally, fatigue is physiologically defined as the loss of voluntary force-producing capacity during exercise. The loss of force-producing capacity can have a peripheral or a central origin. This decline in force or force-generating capacity may originate from various levels of the neural axis, motor cortex, spinal cord to neuromuscular junction, muscle membrane, and metabolism [36]. The nonsignificant changes with muscle fatigue in C4 and CP6 coherence areas have been demonstrated in this study. This is due to the contralateral control of the brain. It is known that, with the right arm lateral raise task in our study, the left brain should be dominant, where the electrodes C3 and CP5 are.

With ELF stimulation, the statistically significant reductions of coherence area with fatigue in C3 and CP5 gamma band disappeared, demonstrating indirectly that muscle ELF stimulation could eliminate the effect caused by the fatigue to a certain extent. Our previous study indicated that the significant difference of power from C3-EEG between fatigue and nonfatigue disappeared with ELF stimulation [16], which suggested the stimulation can influence the activation of cerebral cortex. On the other hand, root mean square and median frequency of EMG were significantly affected by fatigue but not by ELF magnetic stimulation [15]. It is therefore speculated that the EEG-EMG coherence at fatigue was affected by both cortex and muscle. The EEG-EMG coherence changes with ELF stimulation could be affected by proprioceptive afferents impacting on the cortical excitability. The reason for the lack of significance for ‘fatigue' x ‘stimulation' interaction on EEG-EMG coherence could be partially explained by the fact that the measurements of EEG and EMG signals with and without ELF stimulation were from different days. Thus, as shown in Figure 2, the effect of ELF stimulation on EEG-EMG coherence was demonstrated indirectly by the loss of statistical significance of within-session comparisons between fatigue and nonfatigue where both the EEG and EMG signals were recorded simultaneously. One published study observed the increased coherence around 10 Hz for a period up to 250 ms after the transcranial magnetic stimulation [37]. In another study, the increased coherence in the beta band was demonstrated with transcranial magnetic stimulation [38]. The difference in the frequency band between our study and previous ones may be caused by different type of motor tasks and different magnetic stimulations. A published study by Ushiyama et al. [14] has indicated the difference in the modulation patterns of corticomuscular coherence with changing contraction levels between the tibialis anterior and soleus muscles, suggesting that the central nervous system regulates corticomuscular coupling to perform contractions differently between muscles. Regarding the difference between the transcranial magnetic stimulation (to the tibialis anterior muscle) and ELF stimulation (to the deltoid muscle), the key differences are their different stimulation parameters and different impact on proprioceptive afferents. Besides, the local network properties within the sensorimotor cortex differ between these muscles, depending on the physiological muscle compositions and functions.

The present work has some limitations. Firstly, the fatigue status was determined from the subjective feeling of each individual, leading to variations in the calculated EEG-EMG coherence. An objective and consistent criterion for fatigue should be considered to reduce the subjective speculation. Secondly, as a preliminary study, only male subjects were recruited. Both male and female subjects should be recruited in the future to investigate the effect of gender difference. Thirdly, the muscle magnetic stimulation was always applied in the second experiment session. In further study, the order of the sessions with and without magnetic stimulation should be randomized to eliminate the possible bias. Besides, EEG and EMG signals could be recorded simultaneously during the magnetic stimulation to compare the different effects on EEG-EMG coherence during and after the stimulations.

## 5. Conclusions

In conclusion, our study has comprehensively quantified the effects of force, fatigue, and ELF magnetic stimulation on EEG-EMG coherence, demonstrating that corticomuscular coupling changes with fatigue status and ELF magnetic stimulation. Without ELF stimulation, the coherence area in gamma bands from C3 and CP5 electrodes decreased due to muscle fatigue. The application of ELF magnetic stimulation on muscles could eliminate this effect.

## Figures and Tables

**Table 1:** Summary of EEG-EMG coherence area of different electrode, separately for different force loads, for the fatigue/nonfatigue status, and with/without ELF stimulation. The data is presented as mean±SD.

Frequency band	Electrode	Force (kg)	Without ELF stimulation		With ELF stimulation	
			Non-fatigue	Fatigue	Non-fatigue	Fatigue
gamma	C3	0	5.87±0.90	5.55±0.70	5.87±0.86	5.77±0.99
		1	5.82±0.71	5.64±0.70	5.84±1.02	5.67±0.81
		3	5.84±0.77	5.76±0.72	5.78±0.85	5.57±0.69
	CP5	0	5.82±0.72	5.66±0.75	5.86±0.87	5.86±0.87
		1	5.69±0.79	5.54±0.81	5.88±0.87	5.72±0.76
		3	5.95±0.77	5.68±0.76	5.77±0.92	5.75±0.80
	C4	0	5.79±0.77	5.85±0.71	5.78±0.74	5.91±0.78
		1	5.76±0.81	5.72±0.83	5.76±1.05	5.84±0.79
		3	5.82±0.73	5.56±0.73	5.84±0.86	5.92±0.75
	CP6	0	5.76±0.88	5.90±0.68	5.80±0.72	5.75±0.84
		1	5.81±0.87	5.76±0.81	5.79±1.07	5.76±0.85
		3	5.61±0.79	5.65±0.74	5.89±0.76	5.93±0.75

beta	C3	0	4.89±0.75	4.99±0.97	5.02±0.77	4.79±0.98
		1	4.87±0.74	4.99±0.97	4.76±0.86	4.85±0.71
		3	4.84±0.80	4.61±0.77	4.88±0.81	4.66±0.76
	CP5	0	4.79±0.65	4.98±0.91	5.06±0.84	4.85±0.86
		1	4.79±0.78	5.10±0.91	4.79±0.75	4.86±0.91
		3	5.04±0.82	4.57±0.86	4.83±0.85	4.93±0.84
	C4	0	4.89±0.98	4.93±0.96	5.00±0.76	4.82±0.84
		1	4.92±0.89	4.87±0.94	5.01±0.87	4.78±0.89
		3	4.92±0.83	4.81±0.92	5.09±0.72	4.86±0.88
	CP6	0	4.86±0.90	5.00±0.97	4.81±0.69	4.70±0.77
		1	4.81±0.85	4.89±0.85	4.93±0.89	4.58±0.82
		3	4.94±0.88	4.77±1.08	4.94±0.83	4.84±0.81

**Table 2:** Summary of the statistical significance (F/p values) of the effect of different factors (force loads, fatigue/nonfatigue status, and with/without ELF stimulation) on the EEG-EMG coherence area.

Frequency band	Electrode	Force (0/1/3 kg)	Fatigue/non-fatigue	With/without stimulation	Fatigue× Stimulation
gamma	C3	0.07/0.93	7.08/0.006*∗*	0.002/0.96	0.06/0.81
	CP5	0.72/0.46	3.79/0.046*∗*	1.54/0.20	1.02/0.31
	C4	0.33/0.71	0.006/0.94	1.98/0.15	1.78/0.18
	CP6	0.09/0.91	0.05/0.81	1.19/0.27	0.19/0.67

beta	C3	2.33/0.10	0.72/0.40	0.30/0.59	0.66/0.42
	CP5	0.50/0.63	0.003/0.95	0.01/0.91	0.03/0.86
	C4	0.04/0.96	3.15/0.07	0.30/0.58	1.46/0.23
	CP6	0.36/0.70	1.56/0.21	1.30/0.25	2.30/0.13

*∗* p<0.05.

## Data Availability

The datasets generated and analyzed during the current study are not publicly available because the data is unique to our study but are available from the corresponding author on reasonable request.
